# Retrospective survey for sialidase activity in *Mycoplasma pneumoniae *isolates from cases of community-acquired pneumonia

**DOI:** 10.1186/1756-0500-4-195

**Published:** 2011-06-15

**Authors:** Meghan May, Daniel R Brown

**Affiliations:** 1 Department of Biological Sciences, Towson University, Towson MD, USA; 2 Department of Infectious Diseases and Pathology, College of Veterinary Medicine, University of Florida, Gainesville FL, USA

## Abstract

**Background:**

Sialidase is a well-known virulence factor of other respiratory pathogens, but was only recently documented to occur in some species of *Mycoplasma*. The sialidase activity expressed can vary quantitatively among strains within a species of mycoplasma, from undetectable to amounts that correlate positively with strain virulence. Very few isolates of *Mycoplasma pneumoniae *had ever been examined for sialidase activity, so it was unknown whether sialidase may contribute to diseases involving this species.

**Findings:**

No sialidase activity was detected by spectrofluorometric assay of 15 laboratory strains and 91 clinical isolates of *M. pneumoniae *banked over many years from patients having radiologically-confirmed, uncomplicated community-acquired pneumonia.

**Conclusions:**

The annotated genome of strain M129 (GenBank NC_000912, ATCC 29342), also isolated from a patient with pneumonia, accurately represents the absence of sialidase genes from strains of *M. pneumoniae *typically associated with uncomplicated community-acquired pneumonia. A possible involvement of sialidase in neurologic or other extra-respiratory manifestations of *M. pneumoniae *mycoplasmosis remains to be investigated.

## Findings

### Research objective

*Mycoplasma pneumoniae *is primarily associated with interstitial pneumonitis, tracheobronchitis, desquamative bronchitis and pharyngitis, collectively referred to as primary atypical pneumonia (PAP) [1,2]. Mycoplasmosis accounts for 20 - 30% of community-acquired pneumonia (CAP) cases, constituting significant disease and economic burdens in North America and Western Europe. Following its initial association with PAP, other diseases involving *M. pneumoniae *invasion of non-respiratory tissues were reported [Table 1]. Neurologic, dermal, hemotropic, cardiac, arthritic, hepatic, pancreatic, nephritic, and musculoskeletal pathologies have been described[2-12]; many of those diseases occurred secondarily to PAP, either through dissemination of *M. pneumoniae *from the respiratory tract, or following associated autoimmune disorders [3]. Primary extra-respiratory *M. pneumoniae *infections in the absence of PAP, including meningoencephalitis, hepatitis, and pancreatitis, have also been described [13,14], and *M. pneumoniae *has been isolated from the urogenital tract in the absence of clinical signs.

**Table 1:** Diverse outcomes associated with *Mycoplasma pneumoniae *infection^a^

**Aural**	**Dermal**	**Musculoskeletal**
• otitis externa	• bullous exanthema	• reactive arthritis
• otitis media	• cutaneous vasculitis	• rhabdomyolysis
• myringitis	• erythema nodosum	• septic arthritis
Cardiac	• Stevens-Johnson syndrome	Ocular
• arrhythmia	• ulcerative stomatitis	• conjunctivitis
• myocarditis	• urticaria	• iritis
• pericardial effusion	• vesicular rash	• retinitis
• pericarditis	Gastrointestinal	• uveitis
Central nervous system	• hepatitis	Renal
• ataxia	• pancreatitis	• glomerulonephritis
• Bell's palsy	Hematologic	• IgA nephropathy
• choreoathetosis	• aplastic anemia	• interstitial nephritis
• encephalitis	• hemolytic anemia	Respiratory
• encephalomyelitis	• hemophagocytosis	• primary atypical pneumonia
• Guillain-Barre- syndrome	• intravascular coagulation	
• meningoencephalitis	• lymphadenopathy	
• meningitis	• neutropenia	
• paralysis	• neutrophilia	
• polyradiculitis	• septicemia	
• psychosis	• thrombocytopenia	

^a ^The primary disease state generated by *M. pneumoniae *infection is PAP, although many additional clinical manifestations have been reported [2]. Mycoplasmosis due to *M. pneumoniae *affects numerous organ systems by generating primary lesions in non-respiratory tissues or secondary lesions following presumed hematogenous spread from the respiratory tract, or by contributing to autoimmune-related disorders. Differences in bacterial or host phenotypes that explain the diverse clinical outcomes remain essentially unknown.

The majority of studies on clinical aspects of *M. pneumoniae *mycoplasmosis address the diagnosis, treatment and prevention of CAP, while factors predisposing to extra-respiratory diseases remain virtually unexplored. Extracellular "spreading factors" like sialidase are well-known virulence determinants of other pathogenic microorganisms, and are targets for chemotherapy in diseases such as influenza. Sialidase is associated with systemic dissemination during infection with many bacterial species, most notably *Streptococcus pneumoniae *and *Clostridium perfringens*, but such glycosidases were only recently documented to occur in certain species of *Mycoplasma*. The sialidase activity expressed by mycoplasmas can vary significantly among strains within a species, from undetectable to amounts that correlate positively with strain virulence. Very few isolates of *M. pneumoniae *have been examined for sialidase activity, so it was unknown whether sialidase may contribute either to PAP or to extra-respiratory diseases involving this species. To establish the baseline frequency of its occurrance in *M. pneumoniae*, we conducted a retrospective survey for sialidase activity in clinical isolates associated with respiratory mycoplasmosis.

## Methods

Fifteen laboratory strains of *M. pneumoniae*, including the well-known virulent strain PI1428 (American Type Culture Collection accession number 29085, from a patient with PAP), SAD03, SAD05, TW11-4, 1RCH, 9RCH, 11-61, 104.2, 142-48, 256-8, 541-6, 541-29, 1161, 1311 and 15531, plus 91 clinical isolates from cases of radiologically-confirmed interstitial pneumonia were tested. The de-identified clinical isolates had been banked over a period of many years from patients diagnosed with CAP at various locations in the US. They were isolated from four distinct sites in the respiratory tract: 61 were from throat swabs, 4 from nasal swabs, 17 from sputum, and 9 from bronchoalveolar lavage fluid. The latter were considered by clinicians to be invasive because they were from patients hospitalized with severe pneumonia. It was unknown whether any of the patients had been diagnosed as having pneumonia attributable solely to *M. pneumoniae*, or had been treated empirically with viral neuraminidase inhibitors.

Frozen stocks of mycoplasma were passaged one time in SP-4 medium supplemented with 10% w/v glucose and 15% v/v fetal bovine serum at 37°C in ambient air. Sialidase activity of *M. pneumoniae *cells suspended in conditioned medium was assessed using the fluorogenic substrate 2'-(4-methylumbelliferyl)-α-D-*N*-acetylneuraminic acid (MUAN) as previously described [15]. Briefly, 50 μl of stationary phase cultures were added to 50 μl of 0.35% w/v MUAN in 30 mM sodium acetate buffer, pH 4.5. Following 60 min incubation at room temperature, fluorescence at 450 nm was quantitated using a spectrofluorometer (excitation at 350 nm; cutoff filter at 420 nm). The positive controls were *Mycoplasma alligatoris *strain A21JP2^T^, which expresses a cell-associated sialidase [16], and Type VI sialidase purified from *Clostridium perfringens*. The negative controls were *Mycoplasma crocodyli *strain MP145^T^, which does not express any sialidase, and fresh culture medium.

## Results

No sialidase activity was detected in any specimen, thus there was no evidence that the enzyme contributes to PAP involving *M. pneumoniae*. However, the undocumented clinical significance of *M. pneumoniae *isolation from these patients is a recognized weakness of this study. It seems unlikely that potential empiric treatment with viral neuraminidase inhibitors strictly precluded isolation of sialidase-positive strains of *M. pneumoniae *from any of these patients, but this remains to be definitively established.

The ability of certain *M. pneumoniae *strains to cause extra-respiratory mycoplasmosis may require additional cellular functions, which can be genetically acquired *in vivo *from the polymicrobial community within the human host. The presence of bacterial spreading factors such as sialidase might explain the differences in clinical manifestations of infection with certain strains of *M. pneumoniae*. Sialidase is a prominent candidate for this because of its well-documented tendency to be horizontally transferred among bacteria [17]. In addition, quantitative variation of sialidase activity among strains of other pathogenic mycoplasmas ranges from undetectable to extremely high in significant correlation with strain invasiveness [18]. A possible involvement of sialidase in neurologic or other manifestations of invasive disease remains to be investigated, but a bank of those less common specimens will be required to determine whether PAP-associated strains are less likely to express sialidase activity than strains associated with other outcomes of *M. pneumoniae *mycoplasmosis. We conclude that the annotated genome of the well-known virulent strain M129 (GenBank accession number NC_000912), also isolated from a patient with pneumonia (American Type Culture Collection accession number 29342), accurately represents the absence of sialidase genes from strains of *M. pneumoniae *typically associated with uncomplicated PAP.

## Competing interests

The authors declare that they have no competing interests.

## Authors' contributions

MM jointly conceived the study and participated in study design; collected, analyzed and interpreted the data; and drafted the manuscript. DRB jointly conceived the study and participated in study design; reviewed the data analysis and interpretation; and revised the manuscript for important intellectual content. Both authors have read and approved the final version of the manuscript.
